# A Blind Architecture for Signal Combining in Distributed Satellite Communications

**DOI:** 10.3390/s26206409

**Published:** 2026-10-10

**Authors:** Constantin Wimmer, Marco Krondorf, Andreas Knopp, Steffen Bittner

**Affiliations:** 1 Faculty of Engineering, HTWK Leipzig, 04277 Leipzig, Germany; marco.krondorf@htwk-leipzig.de; 2 Munich Center for Space Communications, University of the Bundeswehr Munich, 85579 Neubiberg, Germany; andreas.knopp@unibw.de; 3 Rohde & Schwarz INRADIOS GmbH, 01187 Dresden, Germany

**Keywords:** satellite communications, blind receiver, diversity combining, time-delay synchronization, carrier frequency offset, multi-antenna reception, maximum ratio combining

## Abstract

To meet the increasing demand for high data rates in satellite communications (SATCOM), the requirements in terms of link quality and reliability are also increasing. Reception of SATCOM signals with multiple ground stations and combining them to increase the signal-to-noise ratio (SNR) provides a flexible and robust alternative to larger antenna dish sizes and higher transmit powers. In practical scenarios, the signals received at different ground stations may exhibit different time delays, phase/frequency offsets and noise powers. This might be due to movement of the satellite in low-earth orbit (LEO) and ground stations as well as temperature differences on the ground causing different thermal noise levels. When the transmitting satellite does not cooperate with the ground stations, the central signal processor, e.g., the combiner, has to blindly estimate and compensate for these impairments to achieve optimal combining gain. This paper proposes a blind combiner architecture that integrates time-delay synchronization, frequency synchronization, as well as blind diversity combining to work under practical conditions independently of the signal modulation. The architecture is evaluated in a LEO satellite overflight simulation and validated with real measurements from geostationary satellites. We further derive the theoretical requirements for the accuracy under the different impairments and how these impairments degrade the SNR at the combiner output. The proposed architecture shows a near-optimal SNR performance in both the simulation and measurement results.

## 1. Introduction

SATCOM has seen a significant increase in demand over the last years, driven by its ability to provide global coverage and support of a wide range of applications, such as broadband internet and global navigation [1]. Classical techniques to improve data rates like increased transmit power and increased receive dish size are limited by regulatory constraints and physical limitations. An increase in receiver dish size leads to more wind-load, higher engineering complexity to counter the weight and thus manufacturing cost. Size also highly limits the deployment on mobile platforms [1,2]. These drawbacks motivate the use of multi-antenna receivers with distributed smaller dishes. By coherent combination of the individual signals, the system acts like a virtual larger aperture. This brings a number of advantages over single-antenna reception, such as [1,3,4,5]

- Mitigation of rain-fade with sufficient spatial separation of the receiving antennas, which is increasingly important with the use of higher frequency bands like the Ka-band;
- Simplified deployment on mobile platforms, as smaller dishes are easier to mount and maintain;
- Improved resilience to hardware failures, as the system can continue to operate even if some antennas fail;
- Flexible adaptation of the link budget by changing the number of receive antennas;
- Longer effective overflight times in LEO SATCOM when the receiving antennas are placed along the swath of the satellite trajectory.

Besides SATCOM, the performance of these systems has been demonstrated in applications such as radio astronomy, radar, and sonar [6,7,8]. The concept of coherent signal combination was first introduced in the 1950s for narrowband signals, which led to the formulation of maximum ratio combining (MRC) as the optimal linear combining strategy where the individual signals are weighted according to their channel coefficient and noise power [9]. Distributed arrays additionally require the compensation of time difference of arrival (TDOA) as well as frequency differences of arrival (FDOA). The latter can be caused by transmitter and receiver movement resulting in Doppler shift and different local oscillator (LO) frequencies when a common system clock is not feasible due to large distances between the receiving nodes [10,11]. Studies have shown that the precision of global navigation satellite systems (GNSSs) is not sufficient for TDOA compensation to guarantee high SNR gains at high bandwidths where picosecond accuracy is needed [11]. Considering the above, additional frequency, phase and time synchronization is required at the central processing node (e.g., the combiner). On a system level, a common distinction is made between closed-loop and open-loop implementations. The former relies on feedback from the transmitter to the combiner for calibration which limits the use to cooperative scenarios. Thus, our work focuses on open-loop implementations offering higher flexibility and applicability in non-cooperative scenarios like spectrum monitoring. The downside of open-loop systems is their increased implementation complexity [3]. Since modulation and pilot sequences of the transmit signal are unknown at the combiner, the synchronization and combination have to be performed blindly. Existing work either focuses on coordination of the receiver nodes on the system or hardware level, is restricted to narrowband signals or requires prior modeling of receiver and transmitter positions [10,11,12,13,14,15,16]. In contrast, this paper proposes a unified receive-side combining architecture at the signal processing level without requiring specialized synchronization hardware at the receive antennas. Compared with previous work, this article does not focus on one isolated synchronization or combining block. Instead, it contributes the complete SATCOM-oriented receive-side architecture, the SNR degradation analysis of delay, phase, and weighting errors, and the end-to-end validation of the complete processing chain in LEO simulation and GEO hardware measurements. The contributions of this work are as follows:

- Unified receive-side framework for asynchronous multi-antenna satellite reception. We formulate a unified framework for signal combining with distributed multi-antenna receivers from the SATCOM perspective and derive a joint SNR expression from the channel and compensation parameters. From this expression, we obtain accuracy bounds for delay, phase, and weighting errors.
- Scalable and robust receiver structure: The architecture is scalable at the receiver-network level as it avoids internode communication, shared receiver clocks, and reference-node selection. This also avoids failure when the reference node fails.
- Blind MRC-like combining under realistic receive impairments: The combining stage is designed to approach MRC-like performance without relying on pilots or explicit classical channel estimation and remains effective under arbitrary multicarrier signals and unequal branch noise powers. The former gains importance with the introduction of orthogonal frequency division multiplexing (OFDM) in SATCOM while the latter is relevant for distributed arrays where the receiving nodes can have different hardware or temperatures and thus different noise powers [1,6].
- End-to-end performance evaluation in low-earth-orbit (LEO) overflight scenarios: The complete receive framework is evaluated in a realistic LEO satellite overflight system simulation, enabling assessment of synchronization and combining performance under time-varying impairments. This also hints at the potential of the receiver architecture for other mobile scenarios.
- Real-world validation for geostationary (GEO) satellite reception: The receiver architecture is implemented on a satellite modem and tested with real-world satellite signals from a GEO satellite, demonstrating the practical applicability of the architecture.

Table 1 compares the proposed approach with related methods in terms of their applicability with the impairments and design constraints considered in this work. Open-loop systems which perform over-the-air combining require extensive ranging and synchronization to take into account the time delay and phase shift of the signals while travelling through the channels. Distributed configurable relays perform the necessary phase shift at the relay nodes for coherent superposition at the combiner. A feedback channel from the combiner to the relay nodes is required. Self-calibration methods from radio astronomy heavily rely on known geometry of the site and sky. A common frequency reference is required for the receivers. Algorithms for subspace and multichannel identification rely on simpler models but do not take into account noise or simplify the noise model to equal powers. Methods based on higher-order-statistics (HOS) do not have this drawback but assume non-Gaussian signals. This becomes a problem with increasing number of carriers due to the central limit theorem. Both HOS and subspace methods do not take into account TDOA and FDOA. The National Aeronautics and Space Administration’s (NASA) Deep Space Network (DSN) features the signal-agnostic full-spectrum combining approach and uses a model-aided coarse estimation of TDOA and FDOA such that only slowly varying residual delay and phase errors remain. These are removed using signal correlations. Unequal noise powers are considered in the weighting stage, but not handled blindly. This shows that existing approaches address important parts of distributed coherent reception. However, to the best of the authors’ knowledge, existing methods do not allow blind combining when the receiver nodes are asynchronous and uncoordinated while jointly addressing TDOA/FDOA tracking and signal weighting under unequal branch noise powers without relying on model-based estimates. The proposed architecture fills this gap. The rest of this work is organized as follows: Section 2 introduces the channel and signal model. Section 3 formalizes the problem of signal combining under the influence of the channel effects considered. We derive performance measures for comparison and boundaries for the minimum necessary parameter accuracy. Section 4 describes the proposed receiver architecture and the individual processing steps. An end-to-end performance evaluation through LEO satellite overflight simulation and real-world tests is presented in Section 5. Finally, we conclude this work in Section 6 and give an outlook on future research directions.

## 2. System Model

### 2.1. Scope and Modeling Assumptions

**Figure 1:**
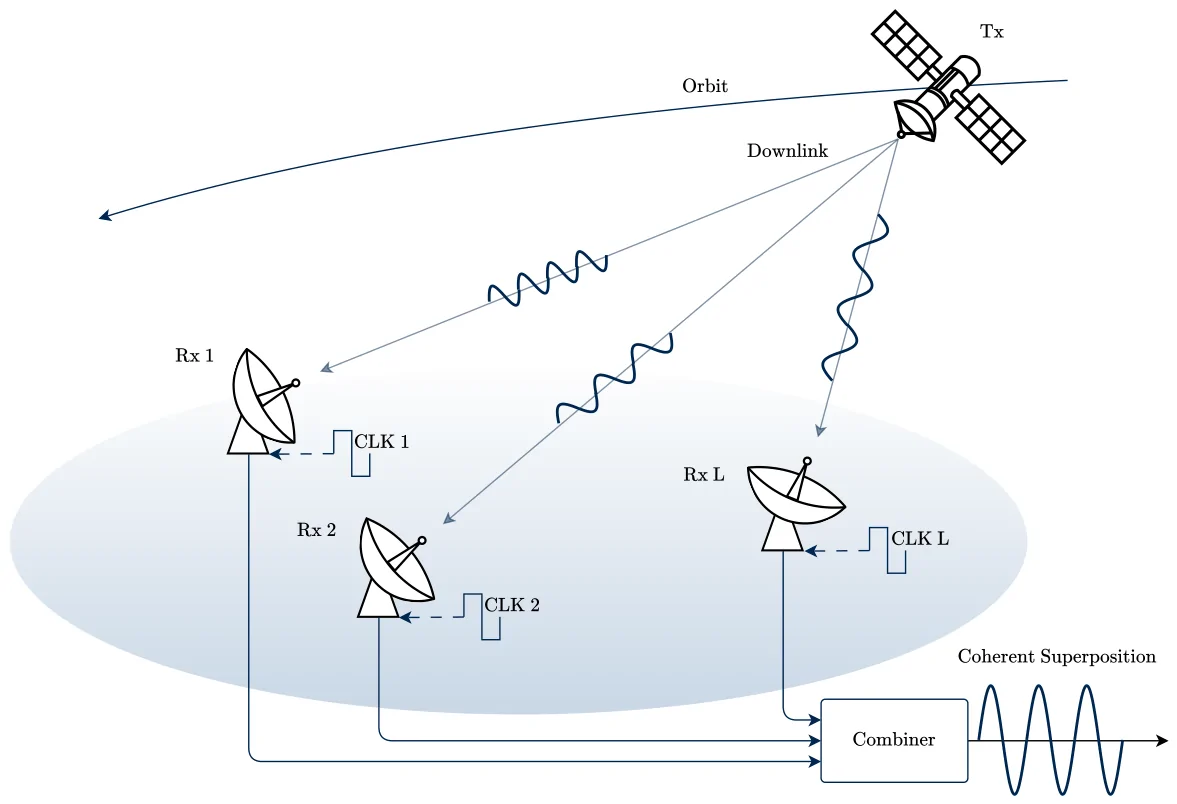
Illustration of the considered system model with a moving satellite transmitting to distributed multi-antenna receive dishes. Independent LOs, Doppler shifts, separation and atmospheric conditions affect the received signals phase, frequency, time differences and amplitude. These effects have to be compensated at the combiner for coherent signal superposition.

We consider a SATCOM scenario as illustrated in Figure 1 with a satellite as the single transmitter and a randomly distributed receive array where the received signals are being fed into a central combiner node. In this context it is feasible to make the following assumptions:

- A common source signal: All receive branches observe the same transmitted signal with different channel effects and noise.
- Flat channels: For fixed SATCOM links with directive ground antennas, the propagation channel is typically dominated by a line-of-sight (LOS) component with additional atmospheric effects like cloud attenuation and tropospheric scintillation. We therefore assume a flat channel impaired by additive white Gaussian noise (AWGN). This assumption is also consistent with the general observation from directional millimeter-wave measurements that narrow beams may substantially reduce the effective delay spread [1,5].
- Asynchronous reception: For distributed arrays with large distances, it is not feasible to have a common system clock. This introduces an unknown clock offset between the receiving antennas downconverters which leads to frequency and phase differences among the receive signals. Additionally, the different locations of the receiving antennas lead to different TDOAs. Changes in temperature and movement of LEO satellites may lead to (slow) time-variations of these impairments.
- Unequal noise powers at the receiving antennas: Distributed arrays may consist of different hardware or be subject to different temperatures which leads to different noise powers at the receiving antennas.

### 2.2. Signal Model

Let s(t)∈C denote an arbitrary complex baseband transmit signal. The signal received at input ℓ∈{1,…,L} in the baseband can be modeled as

(1) $$x_{\ell }\left(t\right)=h_{\ell }s\left(t-\tau_{\ell }\right)e^{j2\pi f_{\ell }t}+n_{\ell }\left(t\right),$$

where

- $h_{\ell }\in C$ is the complex channel coefficient with amplitude *A* and phase $\phi_{\ell }$;
- $\tau_{\ell }\in R$ is the TDOA;
- $f_{\ell }\in R$ is the carrier frequency offset (CFO);
- $n_{\ell }∼\mathrm{CN}\left(0,\sigma_{n,\ell }^{2}\right)$ is the AWGN with noise power $\sigma_{n,\ell }^{2}$;
- *L* is the number of receive branches.

All signals are assumed to be zero-mean and wide-sense stationary. The noise is independent across branches and independent of the signal. For simplicity of modeling, we assume channel coefficient, TDOA and CFO to be only slowly varying compared to the receiver dynamics. In practice, the signals are processed digitally after sampling with a sampling frequency $f_{s}=T_{s}^{-1}$ above the Nyquist rate. Thus, we consider the discrete-time signal model

(2) $$\begin{array}{c} x_{\ell }\left[k\right]=h_{\ell }s\left(\left(k-\theta_{\ell }\right)T_{s}\right)e^{j2\pi \nu_{\ell }k}+n_{\ell }\left[k\right], \end{array}$$

where k∈Z is the discrete-time index and

(3) $$\theta_{\ell }=\tau_{\ell }f_{s},\;\nu_{\ell }=f_{\ell }T_{s}$$

are the discrete-time TDOA and normalized CFO, respectively. For brevity, we omit the sample index *k* in the following when not necessary.

## 3. The Problem of Blind SNR Maximization

### 3.1. Idealized Reference Model

The goal of the combiner is to maximize the output SNR $\gamma_{y}$ without relying on information besides the received signals. To compare the performance of the receiver architecture, we use MRC as the optimal reference combining strategy under the assumption of a perfectly synchronized flat AWGN channel modeled as [35]

(4) $$x_{i}=hs+n$$

with the channel vector

(5) $$h={\left[h_{1}\;h_{2}\;\ldots \;h_{L}\right]}^{T}$$

and the noise vector

(6) $$n={\left[n_{1}\;n_{2}\;\ldots \;n_{L}\right]}^{T}.$$

The output of the idealized combiner

(7) $$y_{i}=w^{H}x_{i}$$

with the weight vector

(8) $$w={\left[w_{1}\;w_{2}\;\ldots \;w_{L}\right]}^{T}$$

and the output SNR

(9) $$\gamma_{y,i}=\sigma_{s}^{2}\frac{w^{H}Hw}{w^{H}\Sigma_{n}w}.$$

Here, $\sigma_{s}^{2}$ is the transmit signal variance,

(10) $$H=hh^{H}$$

is the channel covariance matrix and

(11) $$\Sigma_{n}=diag\left(\sigma_{n,1}^{2},\sigma_{n,2}^{2},\ldots ,\sigma_{n,L}^{2}\right)$$

is the noise covariance matrix. The optimal MRC weight vector is given by

(12) $$\begin{array}{c} w_{*}=w_{0}\Sigma_{n}^{-1}h \end{array}$$

with an arbitrary scaling factor $w_{0}\in C$. Choosing the weights according to Equation (12) the maximum output SNR becomes

(13) $$\begin{array}{cc} {\hat{\gamma }}_{y} & =\sigma_{s}^{2}h^{H}\Sigma_{n}^{-1}h \end{array}$$

(14) $$\begin{array}{cc} \; & =\sum_{\ell =1}^{L}\gamma_{\ell } \end{array}$$

with the branch SNRs

(15) $$\gamma_{\ell }=\sigma_{s}^{2}\frac{|h_{\ell }{|}^{2}}{\sigma_{n,\ell }^{2}}.$$

### 3.2. Performance Measures Under Residual Impairments

In reality, the output signal *y* is the weighted sum of imperfectly synchronized signals $x^{\left(\tilde{\theta },\tilde{\phi }\right)}$

(16) $$y=w^{H}x^{\left(\tilde{\theta },\tilde{\phi }\right)}$$

with the vector of synchronized input signals

(17) $$x^{\left(\tilde{\theta },\tilde{\phi }\right)}={\left[x_{1}^{\left({\tilde{\theta }}_{1},{\tilde{\phi }}_{1}\right)}\;x_{2}^{\left({\tilde{\theta }}_{2},{\tilde{\phi }}_{2}\right)}\;\ldots \;x_{L}^{\left({\tilde{\theta }}_{L},{\tilde{\phi }}_{L}\right)}\right]}^{T}.$$

The *ℓ*-th synchronized signal is given by

(18) $$\begin{array}{c} x_{\ell }^{\left({\tilde{\theta }}_{\ell },{\tilde{\phi }}_{\ell }\right)}\left[k\right]=\left|h_{\ell }\right|s\left(\left(k-\varepsilon_{\theta \ell }\right)T_{s}\right)e^{j(2\pi \varepsilon_{\nu \ell }k+\varepsilon_{\phi \ell })}+n_{\ell }\left[k\right], \end{array}$$

where

(19) $$\varepsilon_{\theta \ell }=\theta_{\ell }-{\tilde{\theta }}_{\ell },\;\varepsilon_{\nu \ell }=\nu_{\ell }-{\tilde{\nu }}_{\ell },\;\varepsilon_{\phi \ell }=\phi_{\ell }-{\tilde{\phi }}_{\ell }$$

are the TDOA, CFO, and phase shift after synchronization with the estimated parameters ${\tilde{\theta }}_{\ell }$, ${\tilde{\nu }}_{\ell }$, and ${\tilde{\phi }}_{\ell }$. To quantify the necessary synchronization accuracy we analyze the output SNR degradation under residual TDOA, frequency, and phase displacement. To model these impairments, we define the cross coherency factor between the impaired transmit signal copies in branches *ℓ* and *m* as

(20) $$\eta_{\ell m}:=\frac{E\left\{u_{\ell }u_{m}^{*}\right\}}{E\left\{|u_{m}{|}^{2}\right\}}$$

where E{·} denotes the expectation operator and

(21) $$u_{\ell }=s\left(\left(k-\varepsilon_{\theta \ell }\right)T_{s}\right)e^{j(2\pi \varepsilon_{\nu \ell }k+\varepsilon_{\phi \ell })}$$

is the impaired transmit signal in branch *ℓ*. Resolving the expectation in (20) gives

(22) $$\begin{array}{cc} \eta_{\ell m} & =\frac{1}{\sigma_{s}^{2}}\left(r_{s}\left(\varepsilon_{\theta \ell }-\varepsilon_{\theta m}\right)e^{j(\varepsilon_{\phi \ell }-\varepsilon_{\phi m})}E\left\{e^{j2\pi (\varepsilon_{\nu \ell }-\varepsilon_{\nu m})k}\right\}\right) \end{array}$$

with the signal autocorrelation function $r_{s}\left(\theta \right)=E\left\{s{\left(\left(k-\theta \right)T_{s}\right)}^{*}s\left(kT_{s}\right)\right\}$. The coherence is then

(23) $$\eta_{\ell m}=\eta_{\phi \ell }\eta_{\phi m}^{*}\eta_{\theta \ell m}$$

where

(24) $$\eta_{\phi \ell }=e^{j\varepsilon_{\phi \ell }}$$

is the phase coherency factor and the delay coherency factor

(25) $$\eta_{\theta \ell m}=\left\{\begin{array}{cc} \frac{r_{s}\left(\varepsilon_{\theta \ell }-\varepsilon_{\theta m}\right)}{\sigma_{s}^{2}}, & \varepsilon_{\nu \ell }=\varepsilon_{\nu m}\\ 0, & \varepsilon_{\nu \ell }\neq \varepsilon_{\nu m} \end{array}\right.$$

which also captures that

$$E\left\{e^{j2\pi (\varepsilon_{\nu \ell }-\varepsilon_{\nu m})k}\right\}=0$$

for $\varepsilon_{\nu \ell }\neq \varepsilon_{\nu m}$. Collecting the delay coherency factors of all pairs yields the delay coherency matrix

(26) $$\eta_{\theta }={\left[\eta_{\theta \ell m}\right]}_{\ell ,m=1}^{L}.$$

Now, expanding the combiner output variance $\sigma_{y}^{2}$ gives

(27) $$\begin{array}{cc} \sigma_{y}^{2}\; & =w^{H}E\left\{x^{\left(\tilde{\theta },\tilde{\phi }\right)}{\left(x^{\left(\tilde{\theta },\tilde{\phi }\right)}\right)}^{H}\right\}w\\ \; & =w^{H}\left(\sigma_{s}^{2}D_{\left|h\right|}\eta_{\phi }\eta_{\theta }\eta_{\phi }^{H}D_{\left|h\right|}^{H}+\Sigma_{n}\right)w \end{array}$$

with the diagonal matrices

(28) $$D_{\left|h\right|}=diag(|h_{1}\left|,\right|h_{2}\left|,\ldots ,\right|h_{L}\left|\right)$$

and

(29) $$\eta_{\phi }=diag\left(\eta_{\phi 1},\eta_{\phi 2},\ldots ,\eta_{\phi L}\right).$$

Splitting that into the signal and noise contribution, we can express the output SNR as

(30) $$\begin{array}{c} \gamma_{y}\left(w,\eta_{\theta },\eta_{\phi }\right)=\sigma_{s}^{2}\frac{w^{H}D_{\left|h\right|}\eta_{\phi }\eta_{\theta }\eta_{\phi }^{H}D_{\left|h\right|}^{H}w}{w^{H}\Sigma_{n}w} \end{array}.$$

This expression captures the coherent combining gain from a coherent power perspective. It also shows that delay and phase displacements and signal weights are coupled in their impact on the output SNR. For tractable design rules, the following subsections therefore derive separate sufficient bounds by isolating one impairment at a time.

### 3.3. Performance Impact of Synchronization Errors

To quantify the effect of the impairments in a convenient way we define the loss factors compared to the idealized reference model. To analyze the SNR degradation under delay and phase displacement, we assume MRC weighting and define

(31) $$\begin{array}{cc} \rho_{\theta \phi } & =\frac{\gamma_{y}\left(w_{*},\eta_{\theta },\eta_{\phi }\right)}{{\hat{\gamma }}_{y}}=\sum_{\ell =1}^{L}\sum_{m=1}^{L}a_{\ell }a_{m}\eta_{\theta \ell m}e^{j(\varepsilon_{\phi \ell }-\varepsilon_{\phi m})} \end{array}$$

using Equations (13) and (30) with the normalized weights

(32) $$a_{\ell }=\frac{\frac{|h_{\ell }{|}^{2}}{\sigma_{n,\ell }^{2}}}{\sum_{m=1}^{L}\frac{|h_{m}{|}^{2}}{\sigma_{n,m}^{2}}}.$$

We obtain a boundary on the necessary phase synchronization accuracy, setting the delay displacement to zero. Then, $\eta_{\theta \ell m}=1$ and

(33) $$\rho_{\phi }={\left|\sum_{\ell =1}^{L}a_{\ell }e^{j\varepsilon_{\phi \ell }}\right|}^{2}.$$

If the pairwise phase mismatch is bounded by $\max_{\ell ,m}\left|\varepsilon_{\phi \ell }-\varepsilon_{\phi m}\right|\leq {\hat{\delta }}_{\phi }$ with ${\hat{\delta }}_{\phi }\in \left[0,\pi \right]$, then we may rotate the coordinate system such that $\varepsilon_{\phi \ell }\in \left[-{\hat{\delta }}_{\phi }/2,{\hat{\delta }}_{\phi }/2\right]$. Since the magnitude is lower bounded by the real part, we can write

(34) $$\begin{array}{c} ℜ\left\{\sum_{\ell =1}^{L}a_{\ell }e^{j\varepsilon_{\phi \ell }}\right\}\geq \sum_{\ell =1}^{L}a_{\ell }\cos\left({\hat{\delta }}_{\phi }/2\right)=\cos\left({\hat{\delta }}_{\phi }/2\right) \end{array}$$

using that $\sum_{\ell =1}^{L}a_{\ell }=1$ and cosine monotonically decreasing in [0,π]. Hence, the lower bound for the SNR loss factor under pairwise phase mismatch is

(35) $$\begin{array}{c} \rho_{\phi }\geq \cos^{2}\left({\hat{\delta }}_{\phi }/2\right). \end{array}$$

The necessary phase synchronization accuracy for a given minimal SNR loss factor $\rho_{\phi }\geq \rho_{\min}$ is

(36) $$\begin{array}{c} {\hat{\delta }}_{\phi }\leq 2\arccos\left(\sqrt{\rho_{\min}}\right) \end{array}.$$

Similarly to the phase, we can derive a boundary on the necessary delay synchronization accuracy setting the phase displacement to zero. Then, $\eta_{\phi \ell }=1$ and

(37) $$\rho_{\theta }=\sum_{\ell =1}^{L}\sum_{m=1}^{L}a_{\ell }a_{m}\eta_{\theta \ell m}.$$

Assume that all pairwise delay mismatches satisfy $|\varepsilon_{\theta \ell }-\varepsilon_{\theta m}|\leq {\hat{\delta }}_{\theta }$ for all ℓ,m and that $r_{s}\left(\tau \right)$ is even and nonincreasing in |τ| on the interval of interest. To obtain a branch-independent worst-case bound, note that each term $r_{s}\left(\varepsilon_{\theta \ell }-\varepsilon_{\theta m}\right)$ becomes smaller as the delay mismatch increases. Therefore, the minimum loss factor is attained when all residual delay shifts are set to the endpoints of the interval

(38) $$\varepsilon_{\theta \ell }\in \left\{-\frac{{\hat{\delta }}_{\theta }}{2},+\frac{{\hat{\delta }}_{\theta }}{2}\right\}.$$

This allows us to group the *L* branches into two sets *A* and *B*. Let

(39) $$P_{A}=\sum_{\ell \in A}a_{\ell },\;P_{B}=\sum_{\ell \in B}a_{\ell }=1-P_{A}.$$

After this grouping, we expand the sum in Equation (37). All same-group pairs have zero relative delay with weight 1. All cross-group pairs have relative delay ${\hat{\delta }}_{\theta }$ and the corresponding weight according to the coherence. Consequently, the loss factor becomes

(40) $$\rho_{\theta }=P_{A}^{2}+P_{B}^{2}+2P_{A}P_{B}\;\frac{r_{s}\left({\hat{\delta }}_{\theta }\right)}{\sigma_{s}^{2}}$$

This expression can be interpreted as an equivalent weighted two-branch system with branch weights $P_{A}$ and $P_{B}$. Rewriting it gives

(41) $$\rho_{\theta }=1-2P_{A}P_{B}\left(1-\frac{r_{s}\left({\hat{\delta }}_{\theta }\right)}{\sigma_{s}^{2}}\right).$$

Since $P_{A}\in \left[0,1\right]$, we can bound independently of the actual weights that

(42) $$P_{A}P_{B}=P_{A}\left(1-P_{A}\right)\leq \frac{1}{4}.$$

It follows that

(43) $$\rho_{\theta }\geq 1-\frac{1}{2}\left(1-\frac{r_{s}\left({\hat{\delta }}_{\theta }\right)}{\sigma_{s}^{2}}\right)$$

Thus, the branch-independent lower bound is

(44) $$\rho_{\theta }\geq \frac{1+\frac{r_{s}\left({\hat{\delta }}_{\theta }\right)}{\sigma_{s}^{2}}}{2}.$$

Therefore, a sufficient condition on the delay accuracy for guaranteeing $\rho_{\theta }\geq \rho_{\min}$ is

(45) $$\begin{array}{c} {\hat{\delta }}_{\theta }\leq r_{s}^{-1}\left(\sigma_{s}^{2}\left(2\rho_{\min}-1\right)\right) \end{array}$$

given that the argument is within the range where $r_{s}$ is invertible unambiguously.

Table 2 compares the maximum allowable delay and phase displacement for a given tolerated SNR loss. For calculations, the raised-cosine correlation function with roll-off 0.35 was used. Given that the maximum SNR gain from combining is 3 dB for two signals and might be even smaller for unequal SNRs, the largest tolerable SNR loss due to synchronization errors should be significantly smaller than 3 dB. Consequently, when designing the synchronization stages one should aim for a maximum residual delay displacement in the order of $0.01T_{s}$ and a maximum residual phase displacement in the order of a few degrees.

**Figure 2:**
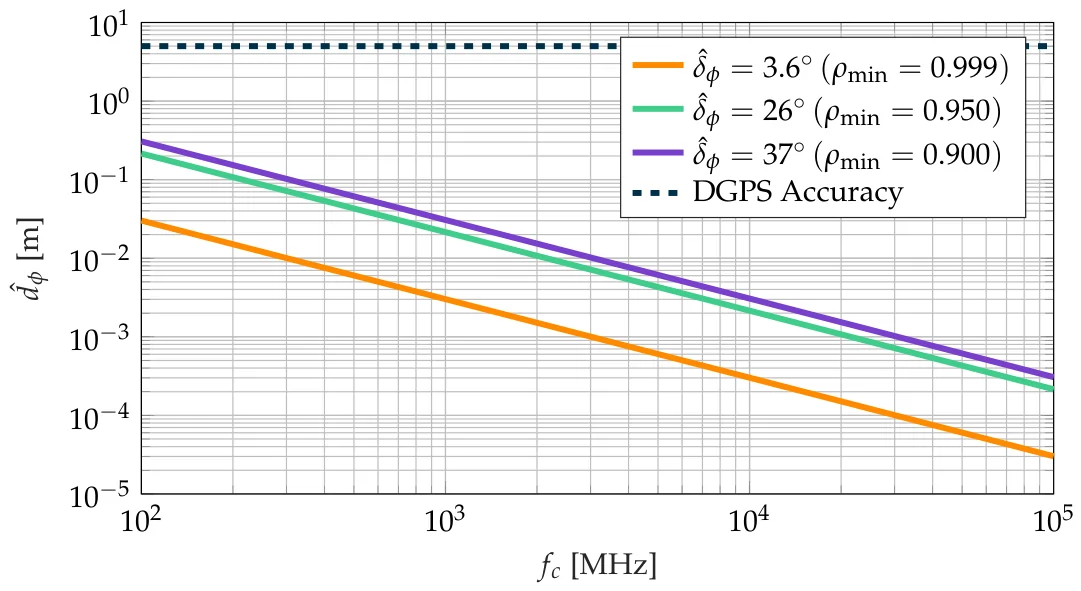
Maximum ranging error for a given tolerated SNR loss from phase displacement over the carrier frequency. For comparison, the accuracy of DGPS ranging is also shown.

This has implications for the design of the synchronization stages. Figure 2 shows the maximum allowed ranging error for a maximum tolerated SNR loss over the carrier frequency $f_{c}$. The maximum ranging error is calculated as

(46) $${\hat{d}}_{\phi }=\frac{{\hat{\delta }}_{\phi }}{2\pi }\frac{c}{f_{c}}$$

where *c* is the speed of light. For comparison, the accuracy of differential Global Positioning System (DGPS) ranging is also shown, which was reported to be approximately 5 m in [36]. It is apparent that the ranging accuracy of DGPS is insufficient to provide the necessary accuracy for low SNR losses $\rho_{\min}>0.9$, even at low carrier frequencies of $f_{\ell }\approx 100$ MHz. This implies that even if position information is available, additional phase synchronization is necessary to achieve high SNR gains.

**Figure 3:**
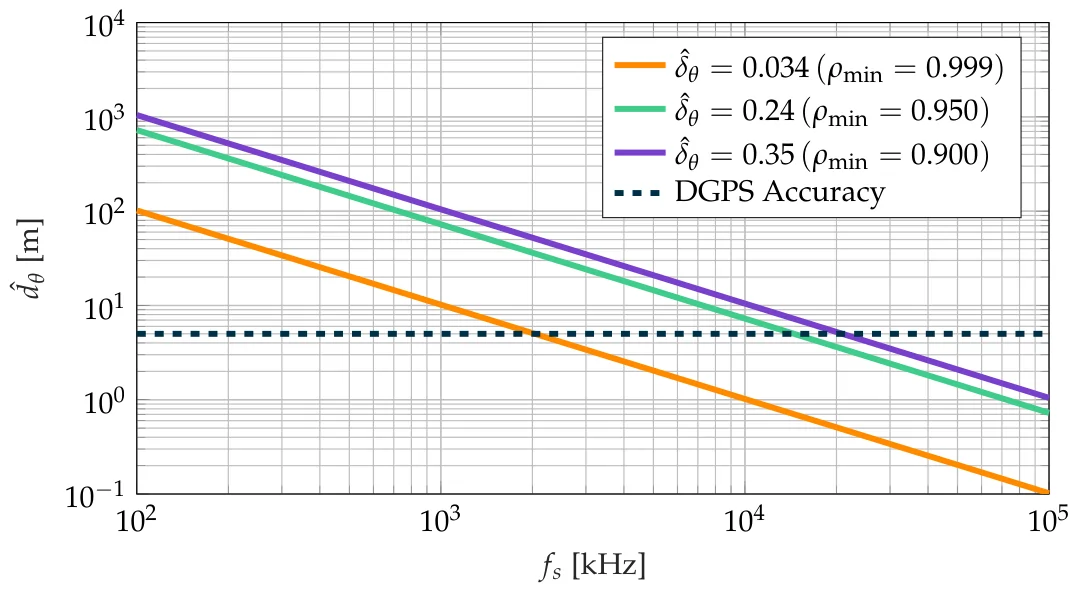
Maximum ranging error for a given tolerated SNR loss from delay displacement over the sampling frequency. For comparison, the accuracy of DGPS ranging is also shown.

Figure 3 shows the maximum allowed ranging error for a maximum tolerated SNR loss over the sampling rate. Here, the maximum ranging error is calculated as

(47) $${\hat{d}}_{\theta }={\hat{\delta }}_{\theta }\frac{c}{f_{s}}.$$

Here, DGPS ranging would be sufficient to provide the necessary accuracy for low SNR losses ($\rho_{\min}>0.999$) for sampling rates $f_{s}<2$ MHz. With increasing $f_{s}$ an additional delay synchronization stage becomes more beneficial. In Section 4.3 and Section 4.4, we will discuss the design of both phase and delay synchronization stages to achieve the necessary accuracy without relying on position information.

### 3.4. Performance Impact of Weighting Errors

Using the maximum output SNR and the SNR for the ideally synchronized case from Equations (9) and (13), we can define the SNR loss factor for the weights as

(48) $$\rho_{w}=\frac{\gamma_{y,i}}{{\hat{\gamma }}_{y}}.$$

Assume that the actual weights satisfy the following relative error model

(49) $$w_{\ell }=c\;w_{*,\ell }\left(1+\delta_{w,\ell }\right)$$

for some common complex scalar c≠0 and relative complex deviation $\delta_{w,\ell }$. Substitution with the whitened variables

(50) $$\tilde{w}=\Sigma_{n}^{1/2}w,\;\tilde{h}=\Sigma_{n}^{-1/2}h,$$

yields the SNR loss factor for the weights as

(51) $$\begin{array}{cc} \rho_{w} & =\frac{|{\tilde{w}}^{H}\tilde{h}{|}^{2}}{∥\tilde{w}{∥}^{2}{∥\tilde{h}∥}^{2}}. \end{array}$$

This is equal to

(52) $$\rho_{w}=\cos^{2}\left(\alpha \right),$$

where α denotes the angle between $\tilde{w}$ and $\tilde{h}$. Under the relative perturbation model with $|\delta_{w,\ell }|\leq {\hat{\delta }}_{w}\in \left[0,1\right]$

(53) $${\tilde{w}}_{\ell }=c\;{\tilde{h}}_{\ell }\left(1+\delta_{w,\ell }\right),$$

the common scalar *c* does not affect the angle, so we may set c=1 and write

(54) $$\tilde{w}=\tilde{h}+e$$

with $e_{\ell }=\delta_{w,\ell }{\tilde{h}}_{\ell }$. Decomposing the perturbation into parallel and orthogonal components relative to $\tilde{h}$ gives

(55) $$e=e_{\|}+e_{⊥}.$$

This forms a right triangle whose adjacent side is $\tilde{h}+e_{\|}$, whose opposite side is $e_{⊥}$, and whose hypotenuse is $\tilde{w}$. Since $\tilde{h}+e_{\|}$ is collinear with $\tilde{h}$, the angle of this triangle is exactly α. Using the definition of $a_{\ell }$ from Equations (32) and (53), the perturbation energy satisfies

(56) $$\frac{{∥e∥}^{2}}{∥\tilde{h}{∥}^{2}}=\sum_{\ell =1}^{L}a_{\ell }{\left|\delta_{w,\ell }\right|}^{2}\leq {\hat{\delta }}_{w}^{2}.$$

Hence,

(57) $$∥e_{⊥}∥\leq ∥e∥\leq {\hat{\delta }}_{w}∥\tilde{h}∥.$$

Interpreting the angular deviation through the orthogonal component then yields

(58) $$\sin\left(\alpha \right)\leq \frac{∥e_{⊥}∥}{∥\tilde{h}∥}\leq {\hat{\delta }}_{w},$$

and therefore

(59) $$\rho_{w}=\cos^{2}\left(\alpha \right)=1-\sin^{2}\left(\alpha \right)\geq 1-{\hat{\delta }}_{w}^{2}.$$

This yields a simple bound on the SNR loss factor

(60) $$\rho_{w}\geq 1-{\hat{\delta }}_{w}^{2}$$

and the necessary accuracy for the weights to guarantee $\rho_{w}\geq \rho_{\min}$ is

(61) $$\begin{array}{c} {\hat{\delta }}_{w}\leq \sqrt{1-\rho_{\min}}. \end{array}$$

Table 3 compares the maximum relative weight perturbation for a given tolerated SNR loss. For the SNR loss to be negligible, one should aim for a maximum relative weight error in the order of a few percent.

## 4. Proposed Blind Coherent Receive Framework

### 4.1. Framework Overview

From the discussion of the optimal weights according to Equation (12) in Section 3 follows that for the given signal model, the combining strategy can be decomposed into three stages:

1. Time Delay Synchronization: The derivation of the optimal weights relies on time synchronized signals. In a wideband scenario, this requires explicit delay compensation.
2. Phase Synchronization: The input signals are weighted by the conjugated channel coefficients so that their phases are aligned. Since the MRC weights require proportionality to the channel coefficients, only any common phase rotation satisfies Equation (12).
3. Signal Weighting: The weights are optimized to maximize the output SNR based on the channel and noise magnitudes. Again, the proportionality constraint allows for any common scaling of the absolute weights without affecting the output SNR.

**Figure 4:**
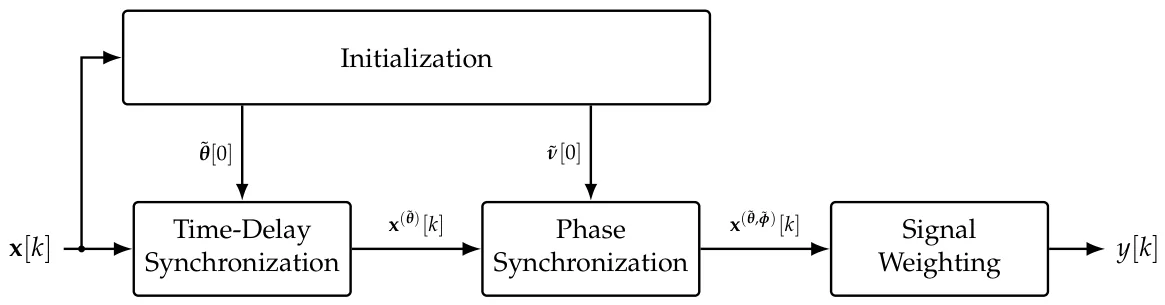
Proposed blind receive architecture for asynchronous multi-antenna satellite reception.

In an environment with changing parameters, the stages must perform continuous tracking of the synchronization and combining parameters. For the delay and phase tracking to work reliably, a coarse initialization for delay $\tilde{\theta }\left[0\right]={\left[{\tilde{\theta }}_{1}\left[0\right]\;{\tilde{\theta }}_{2}\left[0\right]\;\ldots \;{\tilde{\theta }}_{L}\left[0\right]\right]}^{T}$ and frequency $\tilde{\nu }\left[0\right]={\left[{\tilde{\nu }}_{1}\left[0\right]\;{\tilde{\nu }}_{2}\left[0\right]\;\ldots \;{\tilde{\nu }}_{L}\left[0\right]\right]}^{T}$ is required. The proposed receiver architecture is illustrated in the Figure 4. Both synchronization stages get their initial values simultaneously at the start of the combining process. The time-synchronized signals $x^{\left(\tilde{\theta }\right)}\left[k\right]={\left[x_{1}^{\left({\tilde{\theta }}_{1}\right)}\left[k\right]\;x_{2}^{\left({\tilde{\theta }}_{2}\right)}\left[k\right]\;\ldots \;x_{L}^{\left({\tilde{\theta }}_{L}\right)}\left[k\right]\right]}^{T}$ form the input of the phase synchronization stage output $x^{\left(\tilde{\theta },\tilde{\phi }\right)}\left[k\right]$. Since a frequency difference can be viewed as a time-varying phase difference the phase synchronization stage also performs frequency synchronization. The synchronized signals are then combined through weighted summation to form the output y[k]. Table 4 summarizes the interfaces of the stages, their estimation targets as well as the references for a detailed description of the algorithms. The following sections will give a brief overview of the working principles of the stages.

### 4.2. Coarse Initialization via Cross-Ambiguity Function

The synchronization methods for time-delay and phase/frequency are local recursive tracking methods. Their convergence therefore depends on initialization within a suitable attraction region. To provide such initialization from fully asynchronous branch observations, the receiver first performs a coarse pairwise joint TDOA/FDOA acquisition based on the cross-ambiguity function (CAF) [37]. The CAF for a pair of signals $x_{\ell }$ and $x_{m}$ over an acquisition interval of $N_{\mathrm{CAF}}$ samples is

(62) $$A_{\ell m}\left(\theta_{\Delta },\nu_{\Delta }\right)=\sum_{k=0}^{N_{\mathrm{CAF}}-1}x_{\ell }\left[k\right]\;x_{m}^{*}\left[k-\theta_{\Delta }\right]\;e^{-j2\pi \nu_{\Delta }k},$$

where $\theta_{\Delta }$ is the delay hypothesis and $\nu_{\Delta }$ is the frequency-offset hypothesis. The coarse pairwise delay/frequency estimate is obtained by

(63) $$\left({\tilde{\theta }}_{\ell m},{\tilde{\nu }}_{\ell m}\right)=\arg\max_{\theta_{\Delta },\nu_{\Delta }}\left|A_{\ell m}\left(\theta_{\Delta },\nu_{\Delta }\right)\right|.$$

The computation may be optimized by choosing the grid spacing just fine enough to guarantee sufficient correlation. For a maximum path length difference $\Delta d_{\max}$ to test between the branches, the maximum sample delay difference is

(64) $$\theta_{\max}=\left\lceil \frac{f_{s}\Delta d_{\max}}{c}\right\rceil$$

where *c* is the speed of light. A one-sample delay grid is sufficient for coarse initialization in the considered architecture, due to the internal time-delay synchronization stage refining the estimate to sub-sample accuracy. With the one-sample grid spacing and symmetric search range, the number of delay hypotheses is

(65) $$N_{\theta }=2\left\lceil \frac{f_{s}\Delta d_{\max}}{c}\right\rceil +1.$$

For the frequency hypotheses, the grid spacing should be selected such that the residual frequency displacement does not cause severe decorrelation over the CAF interval. A suitable choice is

(66) $$\Delta \nu =\frac{1}{2N_{\mathrm{CAF}}}.$$

With the maximum expected frequency difference $\Delta \nu_{\max}=\Delta f_{\max}/f_{s}$, where $\Delta f_{\max}$ denotes the maximum expected pairwise FDOA in Hz. The number of frequency hypotheses is $N_{\nu }=2\left\lfloor \frac{\Delta \nu_{\max}}{\Delta \nu }\right\rfloor +1$. With Equation (66), the number of hypotheses becomes

(67) $$N_{\nu }=2\left\lfloor 2N_{\mathrm{CAF}}\frac{\Delta f_{\max}}{f_{s}}\right\rfloor +1.$$

A direct CAF implementation therefore has a complexity of $O(L^{2}N_{\mathrm{CAF}}^{2}\left(\Delta d_{\max}\Delta f_{\max}\right)/c)$ ignoring rounding. Since we track a single delay and frequency parameter per signal, we reduce the L(L−1)/2 pairwise estimates to *L* initialization parameters via least-squares reduction under zero mean constraint on the reduced vector. Then, the initialization parameters are

(68) $${\tilde{\theta }}_{\ell }\left[0\right]=\frac{1}{L}\sum_{m=1}^{L}{\tilde{\theta }}_{\ell m}$$

for the delay and

(69) $${\tilde{\nu }}_{\ell }\left[0\right]=\frac{1}{L}\sum_{m=1}^{L}{\tilde{\nu }}_{\ell m}$$

for the frequency respectively.

### 4.3. Adaptive Time-Delay Synchronization

As discussed in Section 3.3, the residual TDOA after synchronization should be in the order of $0.01T_{s}$ for the SNR loss to be negligible. Since the time-delay synchronization is the first stage in the processing chain, it must be able to track the TDOAs under the influence of FDOA. The Cross-Correlation-Explicit Time-Delay Estimation (CCETDE) algorithm from [38] uses the phase invariance of the correlation magnitude to track the TDOA under influence of FDOA. The multichannel extension adapts the delay parameters $\tilde{\theta }={\left[{\tilde{\theta }}_{1}\;{\tilde{\theta }}_{2}\;\ldots \;{\tilde{\theta }}_{L}\right]}^{T}$ through calculation of the short time correlation

(70) $$r_{\ell m}\left({\tilde{\theta }}_{\ell },{\tilde{\theta }}_{m}\right)\left[k\right]=\sum_{p=k-N}^{k+N}{\left(x_{\ell }^{\left(\tilde{\theta }\right)}\left[p\right]\right)}^{*}x_{m}^{\left(\tilde{\theta }\right)}\left[p\right]$$

as well as its derivative with respect to the delay

(71) $$r_{\ell m}^{\prime }\left({\tilde{\theta }}_{\ell },{\tilde{\theta }}_{m}\right)\left[k\right]=\frac{\partial }{\partial {\tilde{\theta }}_{\ell }}r_{\ell m}\left({\tilde{\theta }}_{\ell },{\tilde{\theta }}_{m}\right)\left[k\right].$$

The update rule further requires computations of the delayed output and derivative signals as

(72) $$x_{\ell }^{\left(\tilde{\theta }\right)}\left[k\right]=\sum_{n=-N_{s}}^{N_{s}}x_{\ell }\left[k+n\right]f_{n}\left({\tilde{\theta }}_{\ell }\right),\;x_{\ell }^{\prime (\tilde{\theta })}\left[k\right]=\sum_{n=-N_{s}}^{N_{s}}x_{\ell }\left[k+n\right]f_{n}^{\prime }\left({\tilde{\theta }}_{\ell }\right)$$

with fractional delay filter coefficients $f_{n}\left({\tilde{\theta }}_{\ell }\right)$ and their derivatives $f_{n}^{\prime }\left({\tilde{\theta }}_{\ell }\right)=\partial f_{n}\left({\tilde{\theta }}_{\ell }\right)/\partial {\tilde{\theta }}_{\ell }$. The delay parameters are then updated via gradient ascent with step size $\mu_{\theta }$ as

(73) $${\tilde{\theta }}_{\ell }\left[k+1\right]={\tilde{\theta }}_{\ell }\left[k\right]+\mu_{\theta }\sum_{m=1}^{L}ℜ\left\{r_{\ell m}^{*}\left({\tilde{\theta }}_{\ell },{\tilde{\theta }}_{m}\right)r_{\ell m}^{\prime }\left({\tilde{\theta }}_{\ell },{\tilde{\theta }}_{m}\right)\right\}.$$

**Figure 5:**
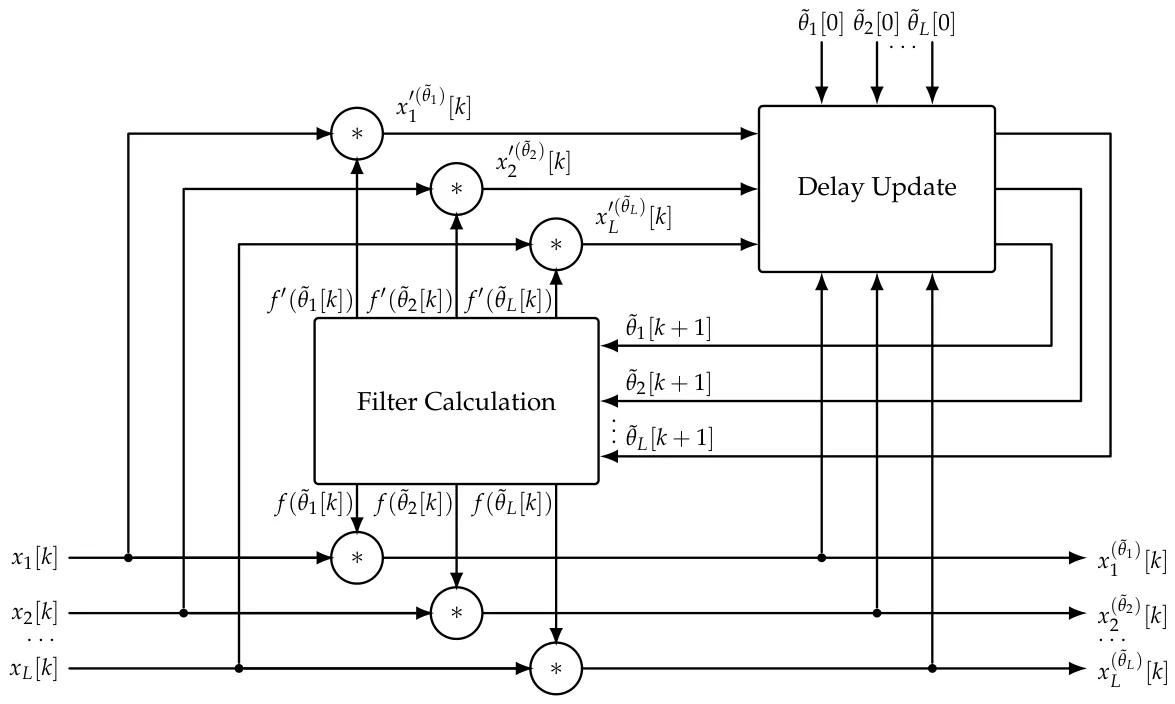
Block diagram of the TDOA synchronization stage. The delay update block outputs updated delay estimations ${\tilde{\theta }}_{\ell }\left[k+1\right]$ which control the branch’s corresponding filters f(·) and $f^{\prime }\left(\cdot \right)$ which the input signal are convolved with (symbol ∗). The filtered signals are then used to calculate the next delay estimations.

Figure 5 shows the feedback loop of the TDOA synchronization stage where the updated delay parameters are fed back to the filter calculation block which also compensates for the delays. The results of the convolutions are then used to calculate the next delay estimates starting with the initial delay values coming from the initialization routine.

**Figure 6:**
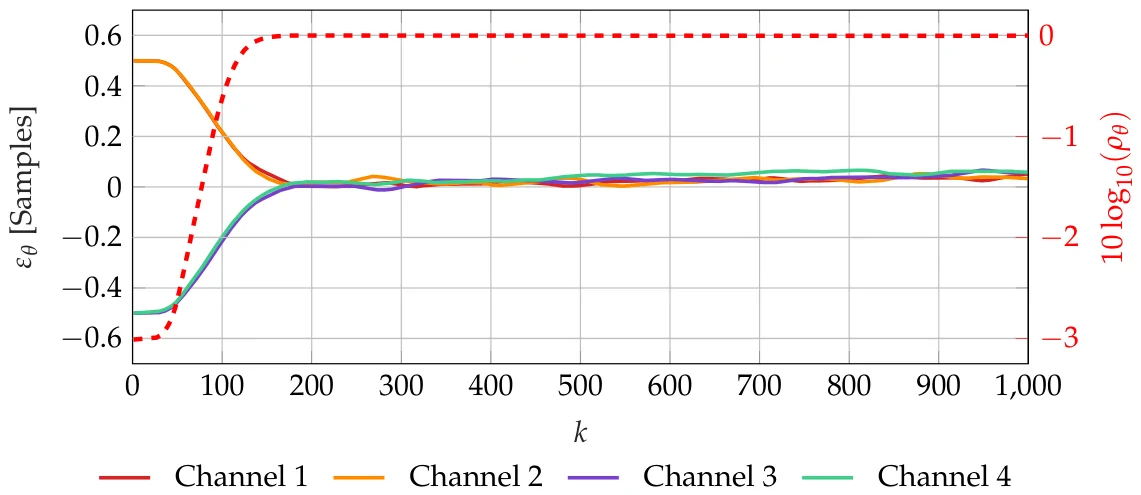
Convergence of the delay synchronization stage with L=4 branches and the corresponding SNR degradation factor. The dashed red line corresponds to the SNR degradation shown on the right axis.

The choice of the filter coefficients $f_{n}\left({\tilde{\theta }}_{\ell }\right)$ largely determines the computational complexity of the algorithm through the length of the summations (72). An efficient choice is the Lagrange interpolator, which also allows for direct calculation of the derivative coefficients $f_{n}^{\prime }\left({\tilde{\theta }}_{\ell }\right)$ using simple lookup tables [41]. This allows for the reduction of the filter length to just 7 coefficients with 80% usable bandwidth. The correlation window length *N* should also be kept small for frequent delay updates and low complexity. Simulations showed that N=1 is sufficient for the algorithm to converge and N=16 offers a good trade-off between convergence speed, noise and FDOA robustness. Figure 6 shows the trajectory of the residual delay shift $\varepsilon_{\theta }$ and the SNR degradation factor over time for $N=16,N_{s}=3,\mu_{\theta }=9\times 10^{-6}$ and normally distributed signals with an SNR of 15 dB. The signal delays are set to ±0.5 and the delay at branch 4 changes with a rate of $2\times 10^{-4}$ per sample. After a short transient of around 150 samples, the delay displacements converged to the same value and the SNR degradation is negligible.

### 4.4. Blind Phase and Frequency Synchronization

At this point, we assume the branch signals to be aligned in time such that the residual delay displacement can be modeled as a small phase rotation:

(74) $$x_{\ell }^{\left({\tilde{\theta }}_{\ell }\right)}\left[k\right]\approx h_{\ell }s\left(kT_{s}\right)e^{j(2\pi \nu_{\ell }k+\phi_{\ell })}+n_{\ell }\left[k\right].$$

The phase-aligned outputs are

(75) $$x_{\ell }^{\left({\tilde{\theta }}_{\ell },{\tilde{\phi }}_{\ell }\right)}=x_{\ell }^{\left({\tilde{\theta }}_{\ell }\right)}e^{-j{\tilde{\phi }}_{\ell }}$$

with phase difference between two branches

(76) $$\Delta \phi_{\ell m}=\arg\left\{h_{\ell }\right\}-\arg\left\{h_{m}\right\}+2\pi \left(\nu_{\ell }-\nu_{m}\right)k-\left({\tilde{\phi }}_{\ell }-{\tilde{\phi }}_{m}\right)+\phi_{n,\ell m}$$

where $\phi_{n,\ell m}$ is a random phase perturbation. A phase alignment algorithm which takes the phase increment $2\pi (\nu_{\ell }-\nu_{m})$ into account performs phase and frequency synchronization simultaneously. The instantaneous phase differences can be obtained from pairwise conjugate multiplications as

(77) $$\Delta \phi_{\ell m}=\arg\left\{x_{\ell }^{\left({\tilde{\theta }}_{\ell },{\tilde{\phi }}_{\ell }\right)}{\left(x_{m}^{\left({\tilde{\theta }}_{m},{\tilde{\phi }}_{m}\right)}\right)}^{*}\right\}.$$

**Figure 7:**
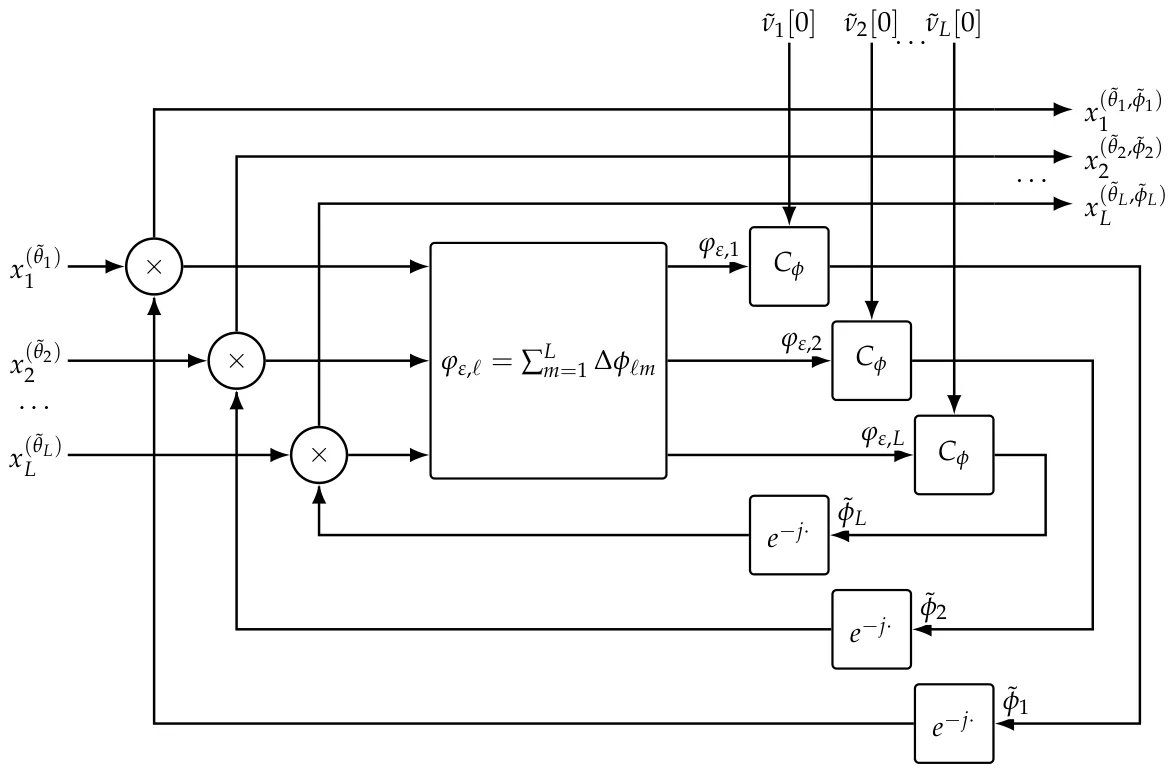
Correlation-coupled feedback control loop for phase and frequency synchronization. The error signal $\varphi_{\varepsilon ,\ell }$ is calculated from the pairwise conjugate multiplications of the input branches which is then fed into the controller $C_{\phi }$ to update the compensation phase ${\tilde{\phi }}_{\ell }$ to align the input signals. The coarse frequency estimates ${\tilde{\nu }}_{\ell }\left[0\right]$ initialize the controller states.

In [39], we proposed to measure the phase mismatch to the other branches as

(78) $$\varphi_{\varepsilon ,\ell }=\sum_{m=1}^{L}\Delta \phi_{\ell m}$$

and use it as an error signal for a controlled feedback loop. Control theory suggests using a second-order integrator for compensation of a constantly changing phase. The controller considered is therefore

(79) $$C_{\phi }\left(z\right)=\frac{\left(z-1\right)K_{P}+K_{I}}{{\left(z-1\right)}^{2}}$$

with proportional gain $K_{P}$, integral gain $K_{I}$ and the *Z*-transform variable *z*. This distinguishes the algorithm from other phase alignment methods like SUMPLE [33], where FDOA is not considered and reference signal-based methods described in [42]. Figure 7 shows the block diagram of the phase and frequency synchronization stage. The phase mismatch $\varphi_{\varepsilon ,\ell }$ is calculated from the pairwise conjugate multiplications and fed into the controller $C_{\phi }$ which updates the compensation phase. The compensation phase ${\tilde{\phi }}_{\ell }$ is then applied to the time-synchronized input signals via complex multiplication. Initial values for the FDOA ${\tilde{\nu }}_{\ell }\left[0\right]$ come from the initialization stage. The algorithm converges to a point where all branches are aligned to the mean of the input phases [39].

**Figure 8:**
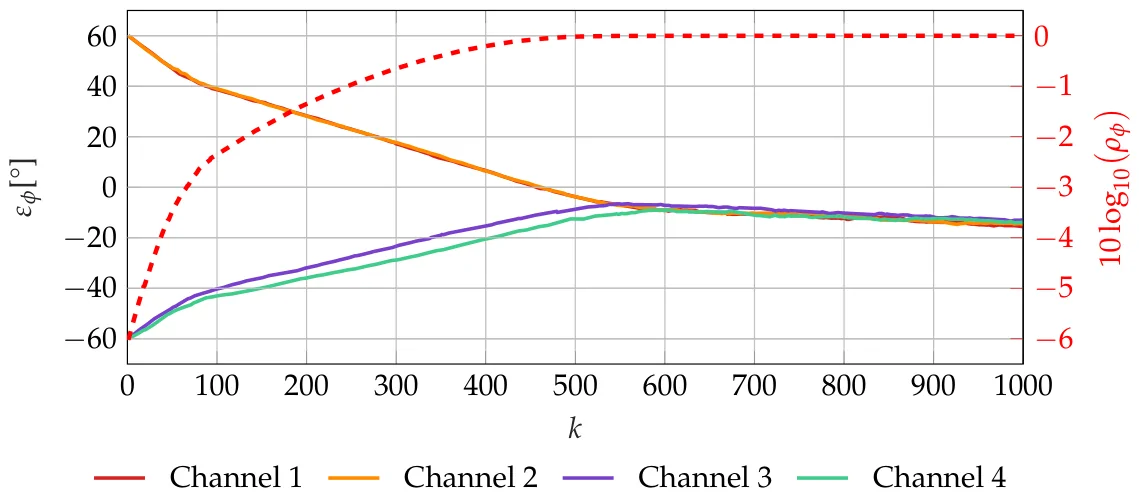
Convergence of the phase and frequency synchronization stage with L=4 branches and the corresponding SNR degradation factor indicated by the dashed red line.

Figure 8 traces the residual phase displacement and SNR degradation trajectories over time where $K_{P}=10^{-3},K_{I}=5\cdot 10^{-7}$ and normally distributed signals with an SNR of 20 dB. The signal phases are set to $\pm 60^{\circ }$ and the frequency at branch 4 changes $10^{-3}$ per sample. The phase displacement converges to the same value after a transient of around 500 samples at which the SNR degradation approaches almost zero decibels.

### 4.5. Blind Noise-Aware Combining

The optimal weights according to Equation (12) must take the channel coefficients and noise variances into account. Blind methods using HOS have been proposed for channel estimation or combining, but they degrade with increasing Gaussianity of the transmitted signal. This renders them less useful for scenarios with high numbers of carriers [24,25]. While other second-order methods exist for blind channel estimation, they typically assume equal noise powers across the branches. In [40], we propose the use of lagged sample covariance to blindly estimate the optimal MRC weights without the assumption of unit noise covariance. At a high level, the weighting scheme exploits the difference between zero-lag and nonzero-lag covariance estimates. Since temporally white receiver noise does not contribute to nonzero integer-lag covariance terms, these statistics can be used to infer MRC weights under unequal branch noise powers. This assumption is exact for temporally white receiver noise. In practical wideband hardware, however, analog and digital filtering may color the receiver noise. Then, the lagged noise covariance is not exactly zero, and the lagged covariance estimate contains a residual noise term that may bias the estimated weights. Possible countermeasures include noise whitening or subtraction of the lagged noise covariance if known. In a scenario with changing channel and noise parameters, the weights must be updated continuously using

(80) $$v\left[k+1\right]=w\left[k\right]+\mu_{w}\tilde{\Delta }w,$$

with a small step size $\mu_{w}>0$ and

(81) $$\Delta w=\left(w^{H}R_{x}^{\left(0\right)}w\right)R_{x}^{\left(1\right)}w-\left(w^{H}R_{x}^{\left(1\right)}w\right)R_{x}^{\left(0\right)}w$$

and

(82) $$\tilde{\Delta }w=\mathrm{sgn}\left(ℜ\{\Delta w\}\right)+j\mathrm{sgn}\left(ℑ\{\Delta w\}\right).$$

The updated vector is normalized as

(83) $$w\left[k+1\right]=\frac{v[k+1]}{{∥v\left[k+1\right]∥}_{2}}$$

for numerical stability. The covariance matrices can be estimated from the received samples as

(84) $$\begin{array}{cc} R_{x}^{\left(0\right)} & \approx \frac{1}{N_{w}}\sum_{k=0}^{N_{w}-1}x\left[k\right]x{\left[k\right]}^{H}, \end{array}$$

(85) $$\begin{array}{cc} R_{x}^{\left(1\right)} & \approx \frac{1}{N_{w}}\sum_{k=1}^{N_{w}}x\left[k\right]x{\left[k-1\right]}^{H}. \end{array}$$

**Figure 9:**
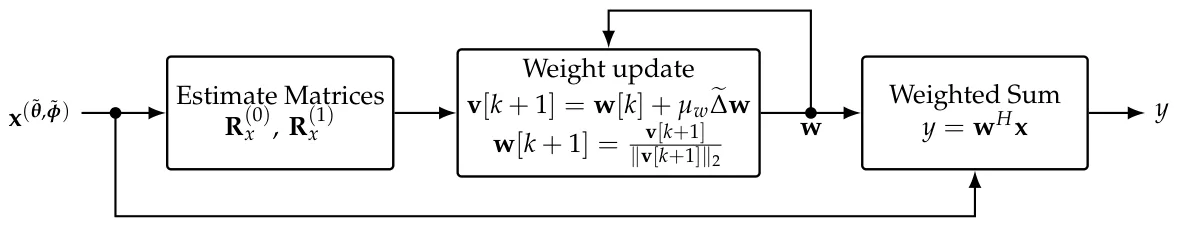
Block diagram of the blind weight tracking and combining stage.

**Figure 10:**
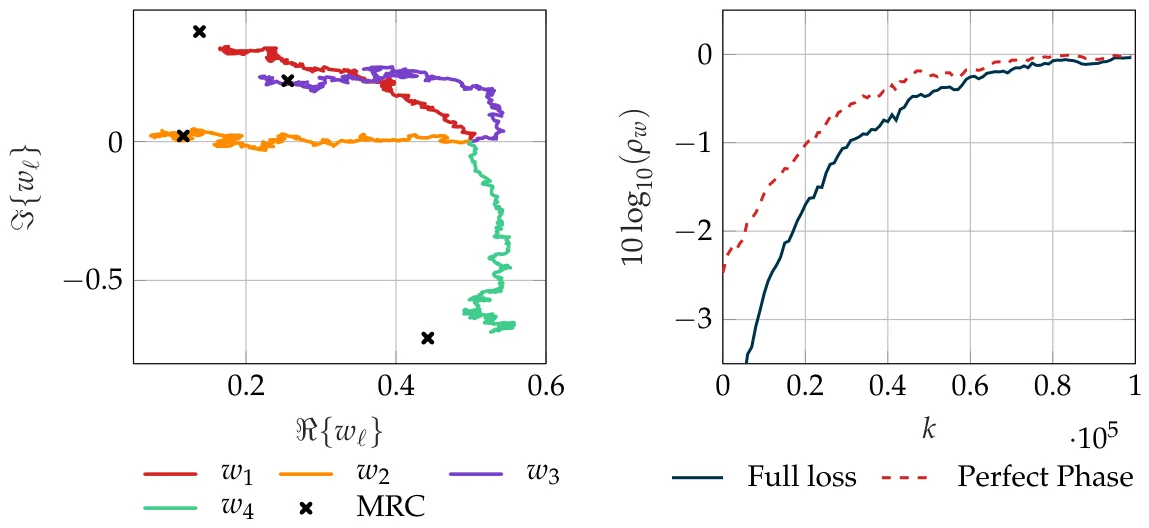
Complex trajectory of the weight vector (**left**) and the corresponding SNR degradation over time (**right**) for phase-aligned and non-phase-aligned signals.

The weight update process is illustrated in Figure 9. For each new set of matrix estimates, the weights are updated and normalized. The weights are then applied to perform the combining through weighted summation of the input branches. Figure 10 shows the trajectory of the weight vector in the complex plane on the left and the corresponding SNR degradation on the right. The summation length is set to $N_{w}=4$ and the step size to $\mu_{w}=5\times 10^{-4}$. The SNRs of the normally distributed input signals range from −3 to 6dB. The weights converge to the MRC values over time and the SNR degradation becomes negligible. The convergence speed can be increased if the phase has been aligned beforehand as indicated by “Perfect Phase”, where only the absolute values of the weights were used to calculate the SNR degradation.

## 5. System Performance Evaluation

### 5.1. Simulation of a LEO Satellite Overflight

For the overflight simulation, the satellite is modeled as moving on a circular orbit at altitude $h_{\mathrm{orb}}$ above a spherical earth. The satellite’s orbit velocity $v_{0}$ follows from the balance of gravitational and centripetal forces. The effective speed $v_{\ell }$ of the ground station relative to the satellite also depends on the earth’s surface speed $v_{E}$ at the specified latitude. To determine the relativistic Doppler shift the projected velocity along the LOS is also needed which depends on the angle $\beta_{\ell }$ between the vertical nadir line and the LOS. A detailed description of the simulation can be found in [43]. The relativistic Doppler shift can be calculated as

(86) $$f_{\ell }=f_{c}\frac{\sqrt{1-\frac{v_{\ell }^{2}}{c^{2}}}}{1-\frac{v_{\ell }^{2}}{c^{2}}\sin\left(\beta_{\ell }\right)}-f_{c}.$$

The time of flight $\tau_{\ell }$ is given by the LOS distance $d_{\ell }$ as

(87) $$\tau_{\ell }=\frac{d_{\ell }}{c},$$

and the received power is assumed to be inversely proportional to the square of the distance $d_{\ell }$ between satellite and receive dish. We normalize to the maximum received power in the nadir position which is proportional to the squared orbit height. The resulting channel gain is

(88) $$h_{\ell }=\frac{h_{\mathrm{orb}}}{d_{\ell }}.$$

**Figure 11:**
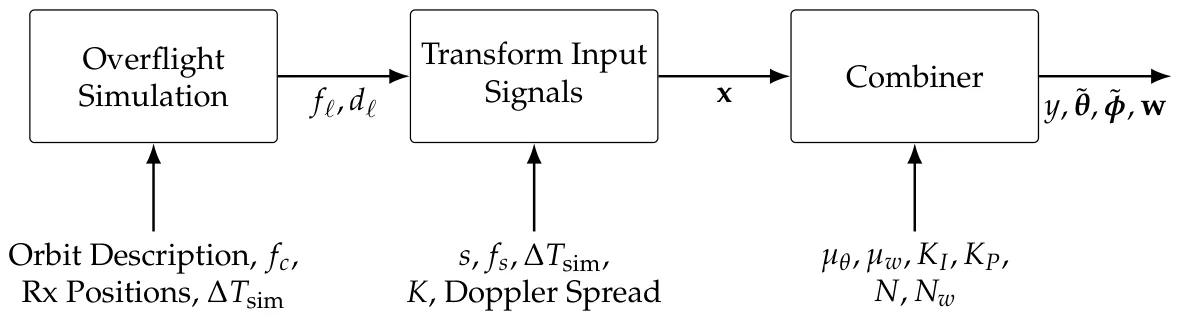
Overview of the simulated combiner behavior during a LEO satellite overflight. The overflight simulation calculates the time-varying Doppler shift and LOS distance to the satellite for each branch. Interpolated to the sample period, these parameters are used to generate the input signals for the combiner.

The LEO simulation runs in a different time base $\Delta T_{\mathrm{sim}}$ than the sampling period of the combiner input signals. Consequently, the overflight parameters require interpolation to the sample period before application to the input signal generation according to Equation (3). The workflow of the overflight simulation is illustrated in Figure 11.

**Figure 12:**
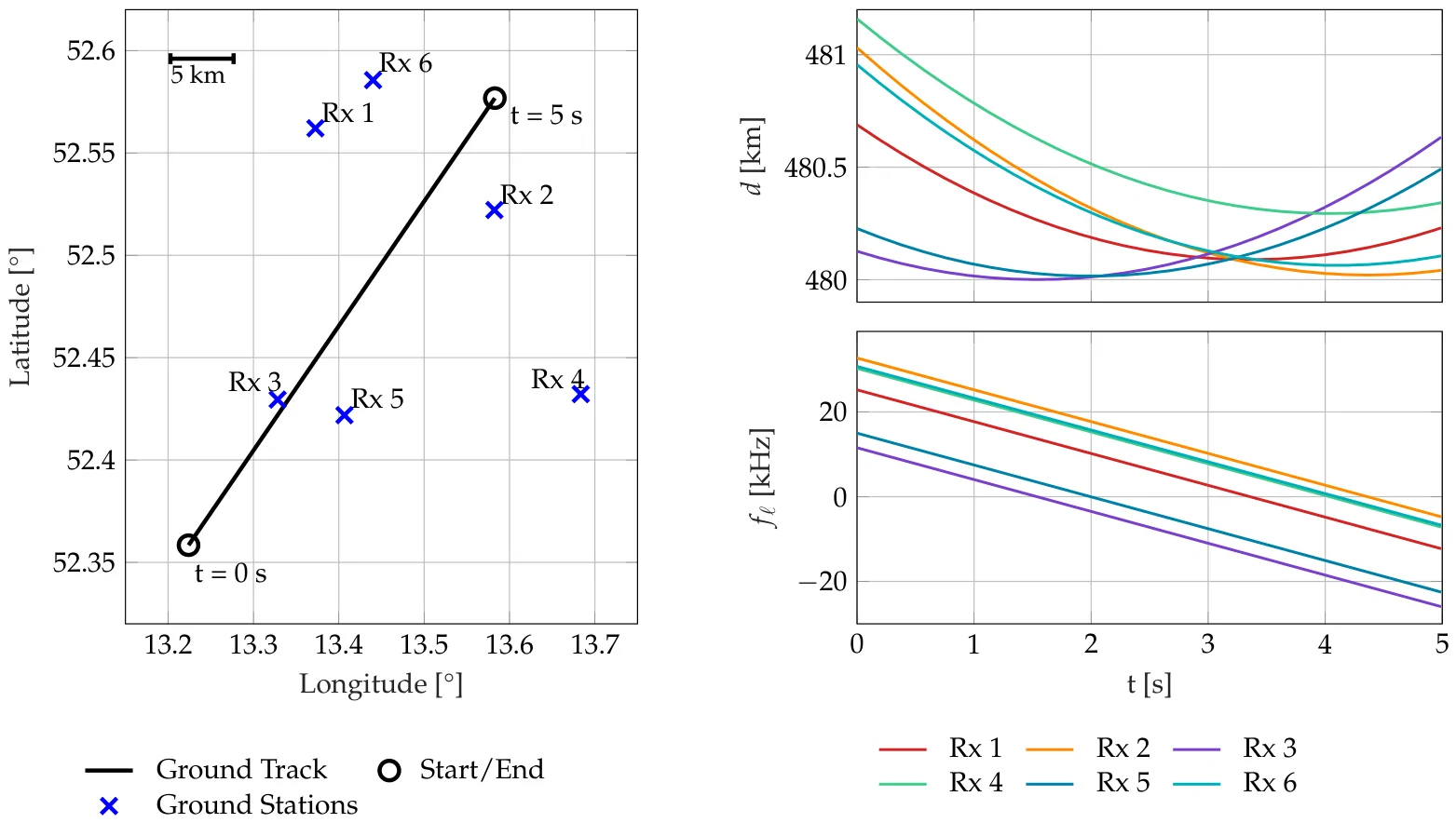
Time-varying signal parameters of the LEO satellite overflight simulation. The top plot shows the LOS distance $d_{\ell }$ and the bottom plot shows the Doppler shift $f_{\ell }$ for each branch.

The simulated scenario consists of six randomly placed ground stations within a square of 20 km side length located at the Berlin area. The satellite enters the area from the south-west at an altitude of 480 km and transmits at the upper Ka-band downlink frequency of 21.2GHz. The left of Figure 12 shows the positions of ground stations and the ground track of the satellite. The right of Figure 12 shows the resulting time-varying LOS distance and Doppler shift for each branch (Rx) when using the parameters of Table 5.

**Figure 13:**
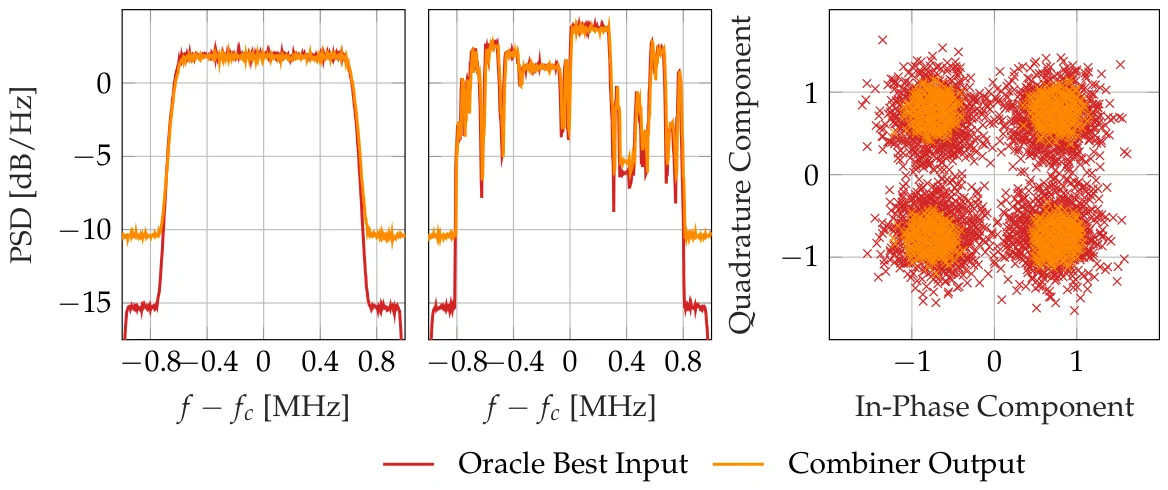
PSDs of the oracle best input signal and the combiner output signal in the QPSK (**left**) and multicarrier case (**middle**). The constellation diagram for the QPSK case (**right**) shows significantly less distortion at the output compared to the input. The SNR improvement is also visible in the lowered noise floor in the PSDs.

To emulate the calculation of the initial values of the combiner, it is held in reset for the first 0.5 s of the simulation. Weights start with equal values and then adapt to the SNR conditions of the branches. The power-spectral-density (PSD) for a constant channel of the best input signal and the output signal of the combiner for the Quadrature Phase-Shift Keying (QPSK) and multicarrier case are shown in Figure 13. In the latter case, every carrier has a random linear modulation ranging from Binary Phase-Shift-Keying (BPSK) up to 128-Quadrature Amplitude Modulation (QAM). A similar reduction of the noise floor is observed for both cases. The constellation diagram for the QPSK case on the right of this figure shows significantly less distortion at the output compared to the input which emphasizes the SNR improvement.

**Figure 14:**
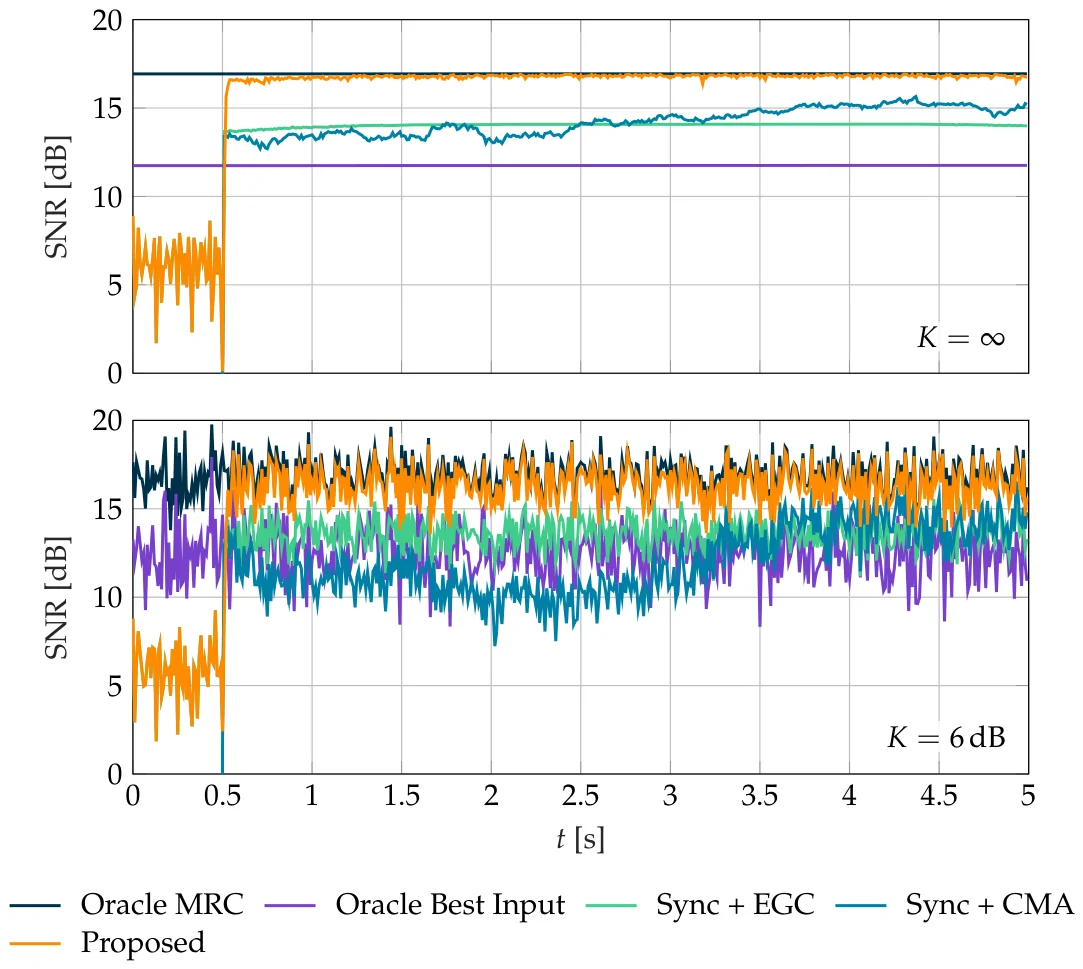
SNR over time during the LEO satellite overflight for the proposed architecture, the theoretical maximum SNR (Oracle MRC), the oracle best input SNR (Oracle Best Input), synchronized EGC, and synchronized CMA. The transmit signal is a multicarrier signal of 100 carriers with random linear modulations.

Figure 14 compares the SNR at the combiner output in the multicarrier case calculated using Equation (30) to the theoretical maximum SNR from MRC using Equation (13) (Oracle MRC). The best branch SNR (Oracle Best Input) $\max_{\ell }\left\{\gamma_{\ell }\left[k\right]\right\}$ is also shown to illustrate the SNR gain. To show the benefit of the proposed noise-aware weighting scheme, the signal weighting and combining is performed using equal weights as well as the constant modulus algorithm (CMA). Since both of these schemes run after the synchronization proposed in this paper, they are labeled as Sync + EGC and Sync + CMA, respectively. The complete architecture of this paper is labeled as Proposed. To show that the combiner can also handle changing channel conditions, the channel is modeled using complex flat Rician fading independent across branches. The Rician fading process is normalized such that the average SNR is the same as in the time-invariant case and the simulation isolates the effect of flat fading. The Doppler spread is set to 50Hz, which models the temporal variation of the channel due to the scattering environment or other channel fluctuations beyond the deterministic Doppler shift, like atmospheric scintillation. Table 6 summarizes the mean SNR and the SNR gain over the best input signal achieved by the different combining schemes in the steady state measured from t=2.5s to the end of the simulation. To support that the proposed architecture does not rely on specific pilots or modulation knowledge, the results are shown for the cases where a single QPSK carrier and a random multicarrier signal are transmitted. It can easily be seen that for the proposed architecture, the results are very similar for both signals transmitted, with a maximum SNR difference of 0.1dB. For a Rice-factor K=∞, the architecture achieves an SNR gain of approximately 0.1dB below the oracle MRC. For K=6dB, the SNR is approximately 0.5dB below the oracle MRC. While the CMA achieves around 0.1dB higher SNR than the proposed architecture in the QPSK case, it significantly falls behind in the multicarrier case by at least 2dB SNR. This has to be expected since the CMA is a HOS method which degrades as the number of carriers and consequently the signal’s Gaussianity increases. It illustrates that CMA can be competitive when its constant-modulus assumption matches the signal, but it is less suitable as a general SATCOM weighting scheme for unknown multicarrier signals. The decreasing performance of the CMA in the multicarrier scenario is also visible in Figure 14 where the SNR for the CMA sometimes falls below the SNR achieved by EGC for both K=∞ and K=6dB.

### 5.2. Physical Implementation and Verification

**Figure 15:**
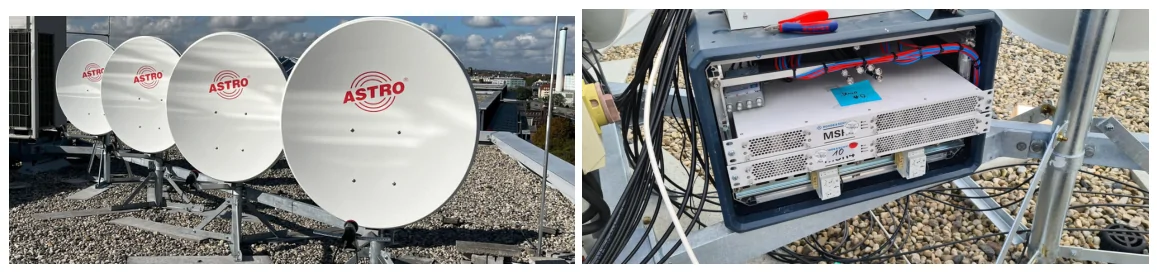
(**Left**) The satellite dishes pointed at a geostationary satellite. (**Right**) The SATCOM modems used for the combiner implementation and DVB demodulation. The modems are equipped with a 4-antenna RF front-end and an FPGA for real-time processing.

For the real-world test, four satellite dishes with a diameter of 1 m are placed on a roof with a spacing of approximately 1.5 m and pointed at the same geostationary satellite. The received signals are fed into a modem equipped with a Field-Programmable Gate Array (FPGA) which the combiner architecture has been implemented on. The modem used in the test setup can be seen in Figure 15 on the right and the satellite dishes on the left. Both dish and its block-down converters use consumer-grade hardware. No reference clock was used to synchronize the system.

**Figure 16:**
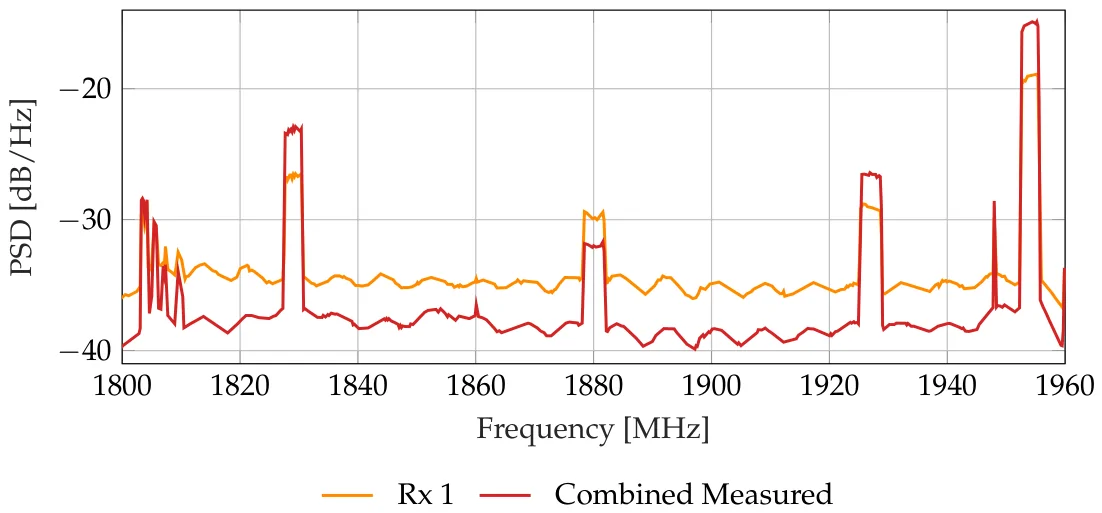
Measured PSDs of the signals at input Rx 1 and the combiner output.

**Figure 17:**
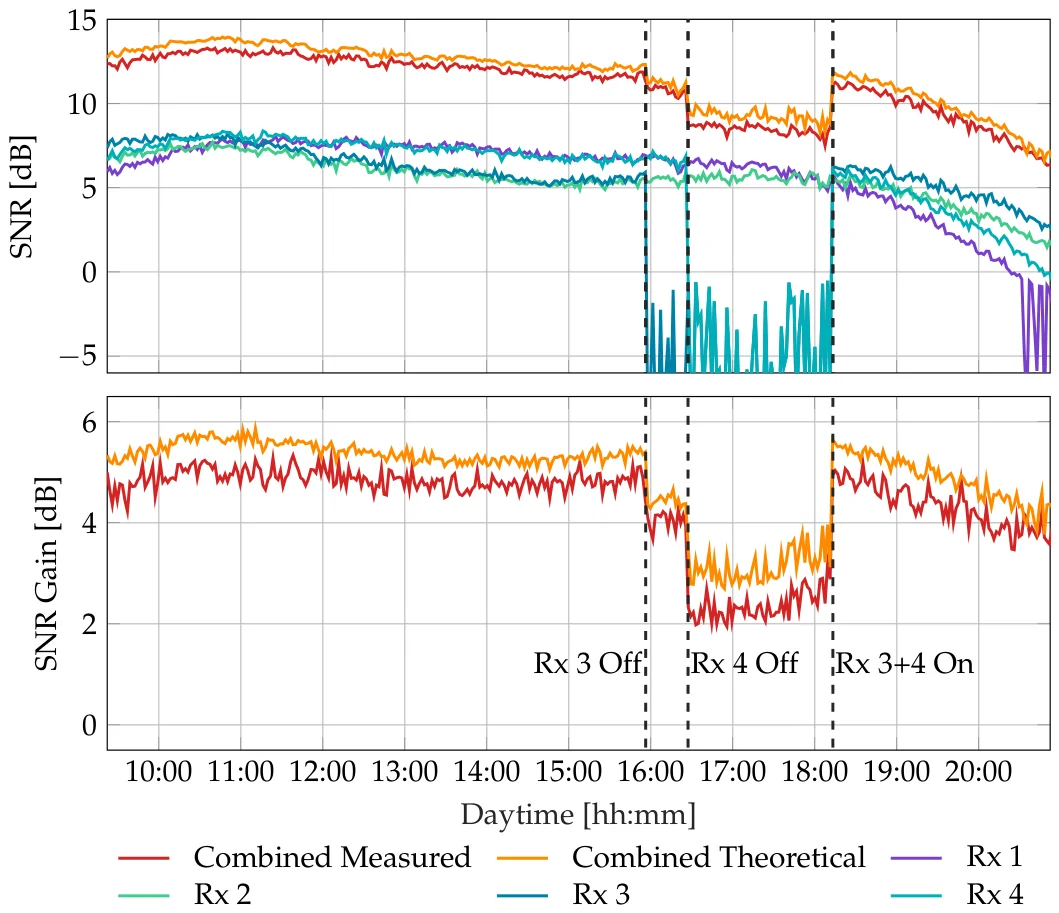
SNR at inputs and output of the combiner and SNR gain over time. The SNR is measured using a DVB receiver tuned to the carrier at around 1930 MHz (see Figure 16). The measured gain is 4.86 dB in the mean with all four inputs active. This translates to an output SNR 0.58 dB below the theoretical maximum obtained using Equation (13).

The signal observed is a multicarrier transponder in the Ku-band with a bandwidth of around 150MHz with a PSD shown in Figure 16. The FPGA runs the combiner at the sample rate of 160MHz. An improvement in carrier-to-noise ratio is clearly visible. The carrier at around 1930MHz is fed into a Digital Video Broadcasting (DVB) receiver which demodulates the signal and measures the SNR from the modulation error. A comparison of the SNRs at the inputs and outputs of the combiner and the subsequent SNR gain is shown in Figure 17. The theoretical values are calculated assuming uncorrelated noise using Equation (13). With four inputs active, the measured gain is 4.86dB. When Rx 3 and Rx 4 are switched off, the measured output SNR is still close to the theoretical maximum value. This allows the DVB receiver to operate without interruption. During the test, the measured output SNR is 0.58dB below the theoretical value in the mean. Besides slight synchronization errors, two hardware-related effects may contribute to this gap. First, a bent-pipe satellite can retransmit uplink noise, which is then observed coherently by all receive branches and sums constructively compared to the uncorrelated noise. Second, the PSD in Figure 16 indicates that the noise floor is not completely flat. This violates the ideal temporally white noise assumption used by the weighting algorithm and the lagged noise-covariance is no longer zero. The resulting residual noise term in the lag-1 covariance $R_{x}^{\left(1\right)}$ may bias the estimated weights.

## 6. Conclusions and Outlook

This paper presented a blind receive-side architecture for coherent signal combining in distributed satellite communications. The proposed architecture integrates adaptive time-delay, phase and frequency synchronization, and blind noise-aware signal weighting. The architecture operates only on the received signals and does not require pilot symbols or knowledge about the signal modulation. It does not require specialized hardware at the ground stations for node coordination through clock sharing or ranging. We derived an expression for the output SNR as a function of pairwise time-delay and phase displacements as well as the signal weights for the given channel coefficients. We used this expression to obtain accuracy requirements for delay and phase synchronization. In addition, a relative perturbation bound was derived for weighting errors. The LEO overflight simulation showed that the proposed architecture approaches the performance of the oracle MRC reference closely under both pure LOS and flat Rician fading conditions. For the considered six-branch scenario, the proposed combiner reached within approximately 0.1dB of the oracle MRC reference in the constant-channel case and within approximately 0.5dB in the flat-fading case. The results were similar for QPSK and multicarrier signals, supporting that the architecture does not rely on a specific signal type. The combiner with the proposed weighting scheme outperforms synchronized CMA by at least 2dB in the multicarrier scenario and performs comparably in the single carrier QPSK scenario. The physical GEO measurement further demonstrated the practical applicability of the architecture with four unsynchronized consumer-grade receive dishes observing an unknown Ku-band multicarrier satellite signal. With all four inputs active, the measured mean SNR gain was 4.86dB. Signal loss did not interrupt the combining process. Overall, the SNR was 0.58dB below the theoretical MRC value computed under the assumption of uncorrelated noise. This gap may be attributed to spatially correlated uplink noise retransmitted by the satellite which constructively sums at the combiner compared to independent noise. Also, colored noise visible at the input violates the assumptions made for the weighting stage. Future work should extend the proposed architecture to frequency-selective channels and explicitly model and mitigate the effect of colored noise.

## Figures and Tables

**Table 1:** Comparison of related combining approaches in their applicability for blind distributed SATCOM reception with asynchronous receivers.

Approach	Unknown Transmit Signals Allowed	Handles Asynchronous or Uncoordinated Nodes	TDOA Tracking	FDOA Tracking	Unequal Noise Powers Allowed	Notes	References
Physical over-the-air	Yes	No	Yes	Yes	No	Requires ranging, time/frequency transfer between coordinated nodes.	[2,3,4,10,11,17,18,19,20]
Distributed configurable Relays	No	No	No	Slow Clock Drift	No	Amplify-and-forward relays, one-bit feedback, packet structure/pilot tone.	[12,13,21]
Higher-order-statistics	Limited	No	No	No	Yes	Generally assumes non-Gaussian or constant-modulus structure. Performance can degrade for multicarrier or OFDM-like signals due to increasing Gaussianity.	[22,23,24,25]
Subspace and multichannel identification	Yes	No	No	No	No	Can identify multichannel systems under simplified assumptions, like equal noise powers or no noise.	[26,27,28,29,30]
Radio-astronomy self-calibration	No	No	No	No	Yes	Estimates parameters with known steering vectors, source geometry or sky models.	[6,15,16,31]
NASA DSN full-spectrum combining	Yes	No	Yes, model aided	Yes, model aided	Not blindly	DSN full-spectrum combining performs coarse TDOA/FDOA model aided. Residual delay and phase errors are obtained by correlation.	[14,32,33,34]
Proposed Architecture	Yes	Yes	Yes	Yes	Yes	Central combiner performs blind signal synchronization and weighting without shared receiver clock or internode communication.	This work

**Table 2:** Allowable maximum delay and phase displacement for a minimum SNR loss factor.

$\rho_{\min}$	$10\log_{10}\left(\rho_{\min}\right)$ [dB]	${\hat{\delta }}_{\theta }$ [Samples]	${\hat{\delta }}_{\phi }$ [^∘^]
0.999	−0.004	0.034	3.624
0.950	−0.223	0.242	25.842
0.900	−0.458	0.349	36.870

**Table 3:** Maximum relative weight perturbation for a minimum SNR loss factor.

$\rho_{\min}$	$10\log_{10}\left(\rho_{\min}\right)$ [dB]	${\hat{\delta }}_{w}$
0.999	−0.004	0.032
0.990	−0.044	0.100
0.900	−0.458	0.316

**Table 4:** Signal interfaces and main parameters of the proposed blind combiner pipeline.

Stage	Input	Output	Estimates	References
Initialization	x[k]	$\tilde{\theta }\left[0\right],\;\tilde{\nu }\left[0\right]$	$\tilde{\theta }\left[0\right],\;\tilde{\nu }\left[0\right]$	[37]
Time-Delay Synchronization	$x\left[k\right],\;\tilde{\theta }\left[0\right]$	$x^{\left(\tilde{\theta }\right)}\left[k\right]$	$\tilde{\theta }\left[k\right]$	[38]
Phase Synchronization	$x^{\left(\tilde{\theta }\right)}\left[k\right],\;\tilde{\nu }\left[0\right]$	$x^{\left(\tilde{\theta },\tilde{\phi }\right)}\left[k\right]$	$\tilde{\phi }\left[k\right],\;\tilde{\nu }\left[k\right]$	[39]
Signal Weighting	$x^{\left(\tilde{\theta },\tilde{\phi }\right)}\left[k\right]$	y[k]	w[k]	[40]

**Table 5:** LEO overflight simulation parameters.

	Parameter		Value
Overflight	Map Center		$52.518^{\circ }$ N, $13.408^{\circ }$ E
	Orbit height	$h_{\mathrm{orb}}$	480km
	Center frequency	$f_{c}$	21.2GHz
	Time step	$\Delta T_{\mathrm{sim}}$	10ms
	Sampling frequency	$f_{s}$	2MHz
	Receiver noise variances	$\sigma_{n,\ell }^{2}$	0.25,0.125,0.5,0.125,1,0.1
	Receive Branches	*L*	6
	Initialization Time		0.5s
Delay Sync.	Learning rate	$\mu_{\theta }$	$1.84\cdot 10^{-7}$
	Correlation Sum Length	*N*	16
	Filter Sum Length	$N_{s}$	8
Phase Sync.	Proportional Gain	$K_{P}$	$5\cdot 10^{-4}$
	Integral Gain	$K_{I}$	$3.13\cdot 10^{-7}$
Combining	Learning rate	$\mu_{w}$	$1\cdot 10^{-3}$
	Correlation Window Length	$N_{w}$	32

**Table 6:** Mean SNR and SNR gain relative to the oracle best input for different transmit signals and combining schemes under flat Rician fading with a Doppler spread of 50Hz.

	Multicarrier		QPSK	
Mean SNR (Gain) [dB]	K=∞	K=6dB	K=∞	K=6dB
Oracle MRC	16.94(5.18)	16.79(4.27)	16.94(5.18)	16.79(4.27)
Oracle Best Input	11.76(0)	12.52(0)	11.76(0)	12.52(0)
Sync + EGC	14.08(2.32)	13.61(1.09)	14.08(2.32)	13.60(1.08)
Sync + CMA	14.81(3.05)	13.08(0.56)	16.88(5.12)	16.41(3.89)
Proposed	16.84(5.08)	16.40(3.88)	16.82(5.06)	16.30(3.78)

## Data Availability

The original contributions presented in this study are included in the article. Further inquiries can be directed to the corresponding author.
